# An Integrative Transcriptomic Analysis of Systemic Juvenile Idiopathic Arthritis for Identifying Potential Genetic Markers and Drug Candidates

**DOI:** 10.3390/ijms22020712

**Published:** 2021-01-12

**Authors:** Daeun Kim, Jaeseung Song, Sora Lee, Junghyun Jung, Wonhee Jang

**Affiliations:** Department of Life Science, Dongguk University-Seoul, Seoul 04620, Korea; daeunkim115@gmail.com (D.K.); jaeseung6455@gmail.com (J.S.); soralee307@gmail.com (S.L.); junghyunjj219@gmail.com (J.J.)

**Keywords:** systemic juvenile idiopathic arthritis, meta-analysis, weighted gene co-expression network analysis (WGCNA), drug repositioning

## Abstract

Systemic juvenile idiopathic arthritis (sJIA) is a rare subtype of juvenile idiopathic arthritis, whose clinical features are systemic fever and rash accompanied by painful joints and inflammation. Even though sJIA has been reported to be an autoinflammatory disorder, its exact pathogenesis remains unclear. In this study, we integrated a meta-analysis with a weighted gene co-expression network analysis (WGCNA) using 5 microarray datasets and an RNA sequencing dataset to understand the interconnection of susceptibility genes for sJIA. Using the integrative analysis, we identified a robust sJIA signature that consisted of 2 co-expressed gene sets comprising 103 up-regulated genes and 25 down-regulated genes in sJIA patients compared with healthy controls. Among the 128 sJIA signature genes, we identified an up-regulated cluster of 11 genes and a down-regulated cluster of 4 genes, which may play key roles in the pathogenesis of sJIA. We then detected 10 bioactive molecules targeting the significant gene clusters as potential novel drug candidates for sJIA using an in silico drug repositioning analysis. These findings suggest that the gene clusters may be potential genetic markers of sJIA and 10 drug candidates can contribute to the development of new therapeutic options for sJIA.

## 1. Introduction

Juvenile idiopathic arthritis (JIA) is one of the most common rheumatic disorders in children, with an incidence of 9.66/million/year in the United Kingdom between 2000–2018 [1]. JIA is characterized by clinical manifestations such as swelling, painful joints, and inflammation [2]. Systemic JIA (sJIA) is a rare form comprising 10–20% of JIA cases and it presents with unique clinical features, such as systemic spiking fevers and rashes, compared with the other subtypes [3]. It was previously reported that dysregulated innate immune responses are prominent in sJIA patients and pro-inflammatory cytokines play crucial roles in the manifestations of sJIA [4]. As such, sJIA is currently considered to be an autoinflammatory disease; however, its complex pathogenesis has not yet been completely understood.

The prognosis of sJIA among other subtypes is known to be the worst in terms of complications and treatment response [5]. Thus, sJIA accounts for a large percentage of mortality occurring in JIA patients, even though it is a rare subtype of JIA [6]. It has been reported that severe complications, such as macrophage activation syndrome (MAS), are associated with the high mortality rate of sJIA [6]. MAS is a life-threatening condition characterized by the excessive activation of T cells and macrophages, resulting in hepatosplenomegaly, lymphadenopathy, cytopenia, coagulopathy, serious liver disease, and kidney failure [7,8]. These fatal complications amplify the need to understand the underlying biological mechanisms of sJIA.

With the advancement of microarray and high-throughput sequencing technologies, previous studies identified differentially expressed genes (DEGs) to unravel genetic factors contributing to the etiology of sJIA [9,10,11]. Several genes, such as *interleukin 1* (*IL-1*), *interleukin 6* (*IL-6*), and *tumor necrosis factor-α* (*TNF-α*) have been identified to be involved in sJIA [9,12]. The majority of DEG studies tend to depend on comprehending the expression profiles of individual genes without accounting for the co-regulation and interactions between genes. However, understanding the intricate interconnection of genes as a unit of a gene set is important in discovering bona fide risk factors. Co-expression network analysis that puts similar gene expression profiles together can be used for determining a set of genes that are simultaneously active in a specific biological process [13]. By applying this analysis to sJIA, thousands of genes can be clustered into co-expression gene sets that may be involved in biologically meaningful pathways.

Along with the transcriptome studies, various treatment options have been developed to manage the clinical symptoms of sJIA, the most commonly used medications being methotrexate, canakinumab, and TNF inhibitors [11,14]. Biological therapies targeting *IL-1* and *IL-6* are also commonly used for sJIA; however, these therapies have limitations as they only aim to alleviate clinical symptoms and, more seriously, may even promote the development of MAS in some patients [15]. Due to these limitations, there is an increasing demand for new drug candidates and new therapeutic options.

In this study, we report the meta-analysis combining multiple microarray datasets from different studies of sJIA for the first time, and the results were confirmed by using an NGS sequencing dataset. Then, co-expression modules that are regarded as clusters of correlated genes were identified using a co-expression network via weighted gene co-expression network analysis (WGCNA) [16]. By integrating the meta-analysis and WGCNA, we were able to obtain robust gene sets made up of positively co-expressed genes as potential genetic markers. Finally, we conducted an in silico drug repositioning analysis to suggest potential drug candidates for sJIA. We believe that our results may provide information on the pathogenesis of sJIA and offer insights into key processes associated with its progression.

## 2. Results

### 2.1. Identification of a Meta-Signature in Human sJIA Datasets

**Figure 1:**
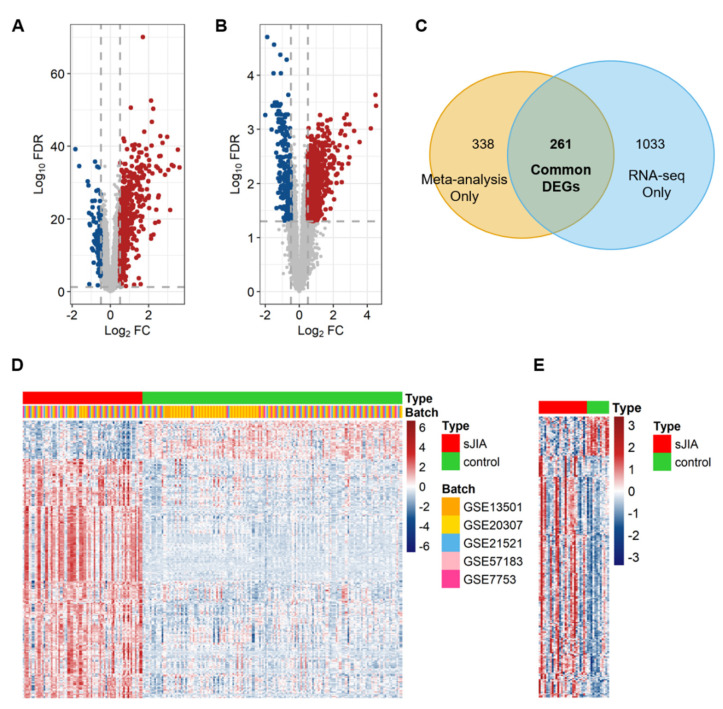
Identification of a meta-signature for systemic juvenile idiopathic arthritis (sJIA). (**A**) A volcano plot showing differentially expressed genes (DEGs) derived from a meta-analysis using microarray datasets. Red and blue dots represent 497 up-regulated genes and 102 down-regulated genes, respectively. The grey dashed vertical and horizontal lines indicate the significance thresholds of log_2_FC (|log_2_FC| > 0.5) and false discovery rate (FDR) (FDR < 0.05), respectively. (**B**) A volcano plot showing DEGs obtained from the RNA-seq dataset. Red and blue dots represent 1041 up-regulated genes and 253 down-regulated genes, respectively. The grey dashes indicate the same significant thresholds as in (**A**). (**C**) A Venn diagram showing the overlap between DEGs identified by the meta-analysis using microarray datasets and the analysis of the RNA-seq dataset. (**D**) A heatmap showing expression patterns of the meta-signature between 81 sJIA and 176 control samples after adjusting for batch effects from five microarray datasets. Rows and columns indicate each gene in the meta-signature and individual samples, respectively. (**E**) A heatmap showing the expression profiles of the meta-signature between 26 sJIA and 12 control samples from the RNA-seq dataset.

To identify robust genetic markers from multiple gene expression datasets, so-called the meta-signature, involved in the pathogenesis of sJIA, a total of 5 microarray datasets, consisting of 81 sJIA patients and 176 healthy control subjects, were collected from the NCBI gene expression omnibus (GEO) (Table 1). Since all of the datasets came from independent studies, batch effects from non-biological variations were adjusted using the sva R package and the total datasets were merged [17,18]. We then performed a principal component analysis (PCA) with the merged dataset to examine whether the tissue source or platform of each dataset resulted in variances of gene expression patterns between samples. The result showed that the gene expression pattern of the merged dataset was mainly classified into sJIA patients and healthy control subjects by disease state, regardless of the tissue source and/or the platform. (Figure S1). We conducted a meta-analysis on the merged microarray dataset and detected DEGs by comparing the expression levels between the sJIA group and the control group. We identified 599 genes as DEGs (false discovery rate, FDR < 0.05), made up of 497 up-regulated genes (log_2_(fold change), log_2_FC > 0.5) and 102 down-regulated genes (log_2_FC < −0.5) (Figure 1A). Comparing the DEGs from individual studies and the meta-analysis revealed that the meta-analysis detected 13 significant genes that were not detected in single studies (Figure S2 and Table S1).

To validate whether the DEGs are also consistently detected in the NGS sequencing data, we selected an RNA sequencing (RNA-seq) dataset with 26 sJIA patients and 12 healthy controls from NCBI GEO (Table 1). We analyzed the RNA-seq dataset and found 1294 DEGs (FDR < 0.05) comprising 1041 up-regulated genes (log_2_FC > 0.5) and 253 down-regulated genes (log_2_FC < −0.5) (Figure 1B). Then, we identified 261 common DEGs that were simultaneously and significantly detected in the meta-analysis and the analysis of the RNA-seq dataset (Figure 1C). Among the 261 DEGs in the merged microarray dataset, 224 genes were up-regulated (log_2_FC > 0.5) and 37 genes were down-regulated (log_2_FC < −0.5) (Figure 1D). For the 261 DEGs in the RNA-seq dataset, 225 genes were up-regulated (log_2_FC > 0.5) and 36 genes were down-regulated (log_2_FC < −0.5) (Figure 1E). Among the 261 genes, we defined 260 genes as the meta-signature of mRNA expression for sJIA (Table S2). One gene was excluded from the 261 genes to improve the consistency of the data because it was regulated differently in the merged microarray dataset and the RNA-seq dataset. Together, we obtained a highly robust meta-signature that could be a representative gene set for sJIA.

**Table 1:** Information on gene expression omnibus (GEO) gene expression datasets used for this study.

Data Type	GEO ID	Tissue Source	Platform	Control/ Disease	Total	PMID
Microarray	GSE21521	Peripheral blood mononuclear cells (PBMCs)	Affymetrix Human Genome U133 Plus 2.0	28/17	45	20576155 [19]
	GSE20307			56/20	76	20662067 [20]
	GSE13501			59/21	80	19565513 [21]
	GSE7753			30/17	47	17968951 [22]
	GSE57183	Whole blood	Illumina HumanHT-12 V4.0 expression beadchip	3/6	9	26267155 [23]
RNA-seq	GSE112057		Illumina HiSeq 2000	12/26	38	29950172 [24]
Total	-	-	-	188/107	295	-

### 2.2. Selecting Co-Expressed Gene Sets from the Meta-Signature as the sJIA Signature

**Figure 2:**
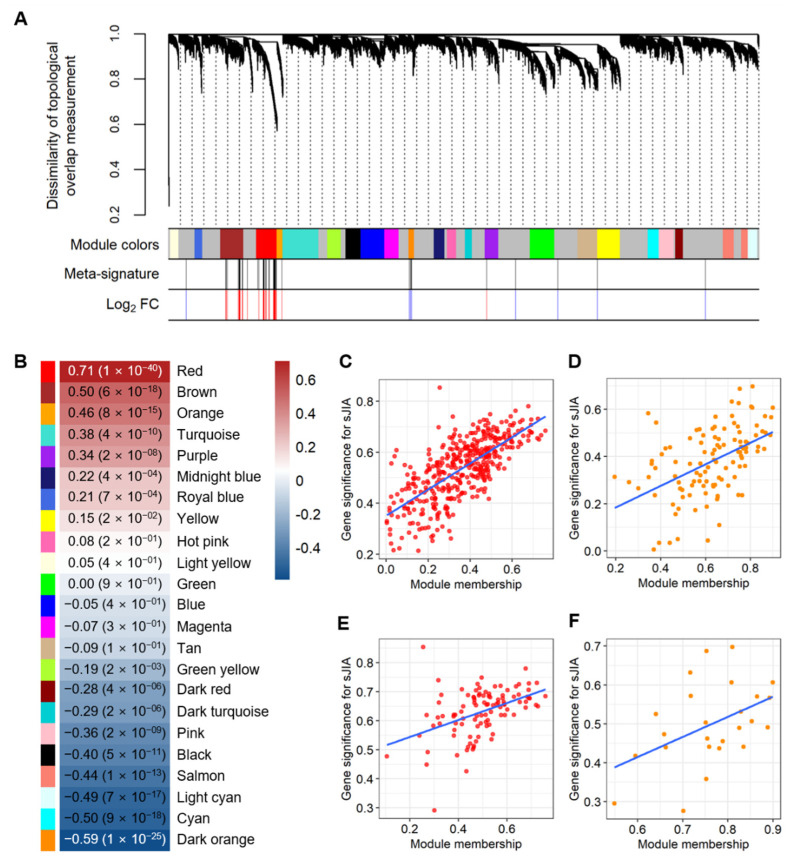
Significant co-expression modules that were enriched with the meta-signature. (**A**) A dendrogram for co-expression modules based on the dissimilarity of topological overlap measurements. Among the three rows at the bottom of the plot, color bars in the first row indicate the randomly assigned colors for the co-expression module names. The orders of the randomly assigned colors for the co-expression module names were light yellow, royal blue, brown, red, orange, turquoise, green-yellow, black, blue, magenta, dark orange, midnight blue, hot pink, dark turquoise, purple, green, tan, yellow, cyan, pink, dark red, salmon, and light cyan. In the second row, each gene in the meta-signature was represented by black lines. Red and blue lines in the final row indicate up- and down-regulation, respectively. (**B**) A heatmap showing the correlations between each module and sJIA. The color blocks shown on the left indicate the randomly selected colors for the module names. Correlation coefficients and the *p*-value for each module are labeled on the heatmap. The range of correlation coefficients is represented by a color bar on the right. (**C**) A scatter plot showing the correlation between the gene significance for sJIA and module membership in the red module, using all of the genes from the module. (**D**) A scatter plot showing the correlation of all of the genes from the dark orange module between gene significance for sJIA and module membership (**E**) A scatter plot showing the correlation between gene significance for sJIA and module membership in the red module, using the meta-signature enriched in the module. (**F**) A scatter plot showing the correlation of the meta-signature enriched in the dark orange module between gene significance for sJIA and module membership. The blue lines in (**C**–**F**) represent the regression lines of the scatter plots.

To comprehend the gene expression profiles in a set of interconnected genes across the sJIA and control samples, we constructed a co-expression network using the merged microarray dataset and WGCNA R package [16]. A soft-thresholding power (β) of 14 was selected for the network construction (scale-free r^2^ = 0.9) (Figure S3). Based on the network, the genes with similar expression profiles were clustered into a co-expression module (r > 0.85). As a result, we identified 23 co-expression modules consisting of genes that had highly-correlated expression patterns (Figure 2A). The genes in each co-expression module are listed in Table S3. To examine whether the clustering patterns of the modules are recapitulated in the RNA-seq dataset, we performed a module preservation analysis using the original dataset as a reference set and the RNA-seq dataset as a test set [25]. The clustering of 22 out of 23 modules from the original dataset was recapitulated in the test set (Z-summary score > 2) (Figure S4). Among the 22 modules, 13 modules were highly preserved in the RNA-seq dataset (Z-summary score > 10). Together, the results suggested that the co-expression gene sets were successfully identified and could be applied across the microarray and RNA-seq datasets of sJIA.

To identify co-expression of the meta-signature that is a robust gene set for sJIA, we mapped each gene from the meta-signature onto the co-expression modules. We then assessed the enrichment of the genes in each module to select the co-expression modules that were highly associated with the meta-signature. The 260 genes of the meta-signature were mapped onto red, brown, dark orange, orange, purple, and yellow modules (Figure 2A). The results of the enrichment analysis between the meta-signature genes and each module showed that most of the meta-signature genes were significantly enriched in the red (two-sided Fisher’s exact test: odds ratio = 23.70 and *p* = 1.04 × 10^−83^), brown (two-sided Fisher’s exact test: odds ratio = 11.39 and *p* = 2.72 × 10^−43^), and dark orange (two-sided Fisher’s exact test: odds ratio = 14.62 and *p* = 1.20 × 10^−19^) modules (Figure 2A and Figure S5). Among the 260 meta-signature genes, the majority of the up-regulated genes were enriched in the red module and the down-regulated genes were enriched in the dark orange module, respectively (Figure 2A). We then performed a correlation analysis between the module eigengene (ME) representing the summary expression profile of each module and sJIA to identify which module had the most significant correlation with sJIA. In line with the data represented in Figure 2A and Figure S5, the results showed that the red and dark orange modules had the most significant positive and negative correlations with sJIA, respectively (red: r = 0.71 and *p* = 1.0 × 10^−40^; dark orange: r = −0.59 and *p* = 1.0 × 10^−25^) (Figure 2B).

To further investigate the association between sJIA and red and dark orange modules, we conducted correlation analyses between the gene significance for sJIA and the module membership, using all of the genes in the red and dark orange modules, respectively. Gene significance and module membership indicated the correlations of a gene to sJIA and the ME of the module, respectively. The genes that were highly associated with sJIA had stronger positive correlations with the respective MEs of the red and dark orange modules (red: r = 0.71 and *p* = 6.69 × 10^−63^; dark orange: r = 0.49 and *p* = 5.70 × 10^−08^) (Figure 2C,D). To validate the representability of the meta-signature in each module, we also performed the abovementioned procedure using the meta-signature genes enriched in the red and dark orange modules. The results from the correlation tests using the meta-signature enriched in each module were consistent with those using all of the genes contained in each module (red: r = 0.45 and *p* = 2.34 × 10^−06^; dark orange: r = 0.45 and *p* = 2.40 × 10^−02^) (Figure 2E,F). Based on these correlation analyses, we revealed that the 103 genes enriched in the red co-expression module and 25 genes enriched in the dark orange co-expression module were significantly correlated with sJIA among the 260 meta-signature genes.

We selected 128 genes consisting of 103 up-regulated genes from the red module and 25 down-regulated genes from the dark orange module as the sJIA signature (Figure 2E,F, and Table S4). Collectively, the meta-signature was enriched in the most highly-correlated co-expression modules with sJIA and was identified as a representative gene set in the modules, indicating that the sJIA signature may have a genetic contribution to sJIA.

### 2.3. Exploring the Biological Pathways Associated with the sJIA Signature

To investigate the biological effects derived from each co-expression module, we conducted functional enrichment analyses on the 23 modules using gene ontology biological process (GO BP) gene sets, then identified GO gene sets significantly associated with each module (*p* < 0.01) (Table S5) [26,27]. The most significantly-associated biological pathways with the modules were presented in the order of module colors in Figure 2A: chemical synaptic transmission (light yellow: *p* = 2.94 × 10^−04^), defense response to virus (royal blue: *p* = 6.31 × 10^−28^), inflammatory response (brown: *p* = 1.62 × 10^−12^), negative regulation of apoptotic process (red: *p* = 6.03 × 10^−06^), platelet degranulation (orange: *p* = 7.72 × 10^−13^), signal transduction (turquoise: *p* = 6.64 × 10^−05^), DNA-templated positive regulation of transcription (green yellow: *p* = 7.98 × 10^−06^), translational initiation (black: *p* = 5.65 × 10^−19^), protein folding (blue: *p* = 1.85 × 10^−05^), SRP-dependent co-translational protein targeting to membrane (magenta: *p* = 5.38 × 10^−08^), cellular defense response (dark orange: *p* = 1.36 × 10^−06^), phospholipid biosynthetic process (midnight blue: *p* = 6.59 × 10^−03^), viral process (hot pink: *p* = 2.39 × 10^−04^), RNA processing (dark turquoise: *p* = 5.83 × 10^−04^), cellular response to zinc ion (purple: *p* = 2.27 × 10^−07^), mitochondrial translational elongation (green: *p* = 5.91 × 10^−13^), intracellular protein transport (tan: *p* = 1.06 × 10^−05^), mitochondrial respiratory chain complex I assembly (yellow: *p* = 8.19 × 10^−05^), cell cycle (cyan: *p* = 1.70 × 10^−04^), DNA-templated transcription (pink: *p* = 2.68 × 10^−04^), B cell proliferation (dark red: *p* = 1.59 × 10^−03^), DNA-templated regulation of transcription (salmon: *p* = 7.76 × 10^−09^), and mRNA processing (light cyan: *p* = 1.07 × 10^−10^).

**Figure 3:**
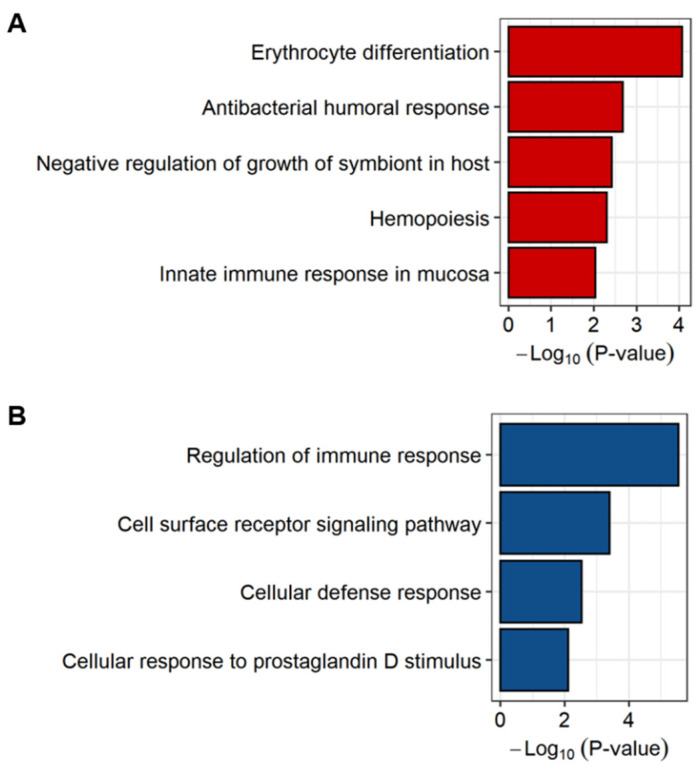
Functional enrichment analysis of the sJIA signature. The sJIA signature used as an input to the database for annotation, visualization, and integrated discovery (DAVID) is composed of 103 up- and 25 down-regulated genes. (**A**) A barplot showing biological pathways significantly associated with the up-regulated genes of the sJIA signature (*p* < 0.01). (**B**) A barplot showing biological processes significantly associated with the down-regulated genes of the sJIA signature (*p* < 0.01).

Among them, the most highly-correlated modules with sJIA, the red and dark orange modules, were respectively involved in negative regulation of apoptotic process and cellular defense response, which may be related to immune responses. We then conducted a functional annotation of the sJIA signature to further focus on its biological effects. The analysis was performed on 103 up-regulated genes and 25 down-regulated genes, respectively. A total of 5 GO terms were associated with the up-regulated genes of the sJIA signature (*p* < 0.01) (Figure 3A and Table S6). Three out of 5 biological pathways, antibacterial humoral response, negative regulation of growth of symbiont in the host, and innate immune response in the mucosa, were involved in innate immune responses that have been emphasized as representative features of sJIA. The other 2 terms were involved in erythrocyte differentiation and hemopoiesis. Erythrocyte differentiation is the process of development from erythropoietic stem cells to mature red blood cells and hemopoiesis is the formation of cellular components of the blood in myeloid or lymphatic tissue [28,29]. Even though the major function of red blood cells is carrying oxygen, they are also involved in inflammatory processes that are the characteristics of sJIA [30].

For the functional annotation of down-regulated genes, a total of 4 GO terms, regulation of immune response, cell surface receptor signaling pathway, cellular defense response, and cellular response to prostaglandin D stimulus were associated with the down-regulated genes of the sJIA signature (*p* < 0.01) (Figure 3B and Table S7). Most pathways, such as the regulation of immune responses, cell surface receptor signaling pathway, and cellular defense response, are involved in the dysregulation of cellular responses. These results were mainly attributed to the genes encoding killer cell lectin-like receptor (KLR) proteins among the 25 down-regulated genes (Table S7). Put and colleagues reported that the expression levels of immune-regulating genes were decreased in sJIA patients and natural killer cells exhibited decreased expression of *KLRG1* [7]. Together, our results suggest that innate immune responses and dysregulation of cell signaling pathways in natural killer cells may be significantly involved in the pathogenesis of sJIA.

### 2.4. Identification of Key Genes among the sJIA Signature by Network Analysis

**Figure 4:**
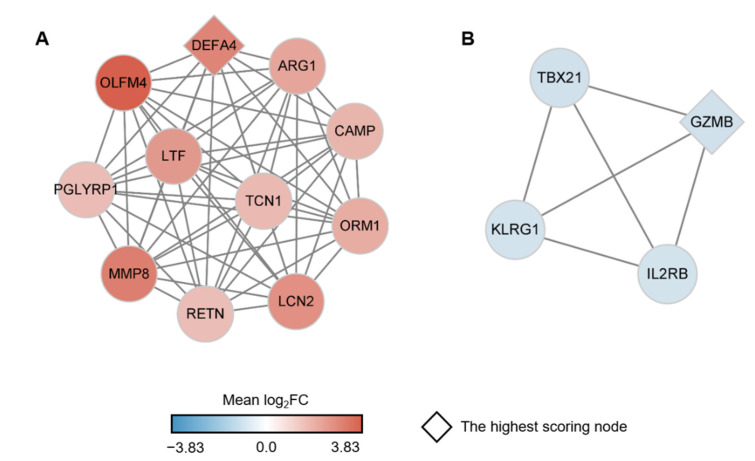
The significant gene clusters within the PPI network comprising the sJIA signature. (**A**) A gene cluster with the highest rank score, comprising the up-regulated genes of the sJIA signature. (**B**) A gene cluster with the highest rank score made up of down-regulated genes of the sJIA signature. The range of the mean log_2_FC values of the nodes is represented as a color bar at the bottom. Red and blue color nodes correspond to up- and down-regulated genes. The seed nodes are represented by rhombi.

To identify key player genes within the sJIA signature, we constructed a protein-protein interaction (PPI) network using 128 genes of the sJIA signature as nodes. After filtering the nodes using the search tool for the retrieval of interacting genes (STRING), the remaining 66 genes were incorporated into the final network (Figure S6 and Table S8) [31]. As expected, we observed that the majority of the genes were linked to the other genes included in the same module. Then, highly-interconnected genes were detected as clusters based on the topology of the PPI network using molecular complex detection (MCODE) [32]. We identified the most significant cluster having the highest rank score generated by MCODE in the up-regulated genes of the red module and the down-regulated genes of the dark orange module, respectively. The cluster with the highest rank score of 11 from the red module is comprised of 11 up-regulated genes (Figure 4A). Among these genes, the highest-scoring node, also known as the seed node, was *defensin alpha 4* (*DEFA4*; mean log_2_FC = 2.88 and total degree = 14) and the gene with the highest log_2_FC value was *olfactomedin 4* (*OLFM4*; mean log_2_FC = 3.82 and total degree = 10). *DEFA4* encodes a member of defensins thought to be associated with host defense and *OLFM4* encodes an anti-apoptotic factor that promotes tumor growth [33,34]. Since the 2 genes were reported as up-regulated DEGs of sJIA by Brachat et al., we were able to confirm that our result conformed with the previous research [11]. Even though the majority of the components in the cluster were mentioned as sJIA- or JIA-related genes, they were rarely highlighted as genetic markers for sJIA in previous studies [4,11,22,35,36,37]. Especially, *orosomucoid 1* (*ORM1*; mean log_2_FC = 1.91 and total degree = 10) and *transcobalamin 1* (*TCN1*; mean log_2_FC = 1.56 and total degree = 10) were not mentioned in previous studies regarding sJIA, as far as we know. Together, we identified 2 novel genes, *ORM1* and *TCN1,* that are involved with sJIA and the up-regulated gene cluster including these 2 genes may possibly be a set of actual genetic markers and druggable targets for sJIA (Figure 4A).

The cluster with the highest rank score of 4 from the dark orange module comprises 4 down-regulated genes (Figure 4B). *Granzyme B* (*GZMB*; mean log_2_FC = −0.83 and total degree = 5) that encodes part of the peptidase S1 family of serine proteases was the seed node and the gene with the most negative log_2_FC value in the cluster [38]. It was reported by Zhou et al. that the expression level of *GZMB* was lower in sJIA patients compared with healthy subjects [39]. *KLRG1* (mean log_2_FC = −0.76 and total degree = 3) was also reported as a down-regulated gene in sJIA [40]. *Interleukin 2 receptor subunit beta* (*IL2RB*; mean log_2_FC = −0.77 and total degree = 3) was previously proposed to be significantly associated with JIA, and a knock-out mice study showed that the absence of functional *IL2RB* leads to severe arthritis [41]. Based on these previous studies, we were able to confirm that our study reliably detected down-regulated genes of sJIA. Notably, *T-box transcription factor 21* (*TBX21*; mean log_2_FC = −0.82 and total degree = 3) has not previously been mentioned as a significant gene for sJIA, to the best of our knowledge. Together, we found novel risk genes that may be used as therapeutic targets for sJIA and these significantly up- and down-regulated gene clusters may be involved with the pathogenesis of sJIA.

### 2.5. Identification of in Silico Drug Candidates for sJIA

To discover drug candidates targeting the significant up- and down-regulated gene clusters, we performed a drug repositioning analysis using more than 7000 drug signatures in the database of connectivity map (CMap) [42]. The results showed that a total of 10 bioactive molecules, *N*-(4-aminobutyl)-5-chloronaphthalene-2-sulfonamide (W-13), ketanserin, gelsemine, lobeline, colforsin, suprofen, lycorine, vincamine, racecadotril, and streptozocin, were identified as potential drug candidates for sJIA (*p* < 0.05) (Table 2). The drug candidate with the highest enrichment score was W-13 (enrichment score = 0.95 and *p* = 5.77 × 10^−03^), which acts as a potent antagonist of calmodulin and is widely used to examine Ca^2+^/calmodulin-regulated enzyme activities [43]. Audran et al. reported that calmodulin is involved in rheumatoid arthritis and could be a therapeutic target in inflammatory diseases [44]. Among the 10 potential drug candidates, the majority of the candidates, such as ketanserin, gelsemine, lobeline, colforsin, suprofen, lycorine, and vincamine, are known to be anti-inflammatory molecules. The topical application of ketanserin (enrichment score = 0.79 and *p* = 3.94 × 10^−03^) was previously suggested as a promising approach to relieve inflammation in arthritis [45]. Gelsemine (enrichment score = 0.77 and *p* = 5.61× 10^−03^), lobeline (enrichment score = 0.74 and *p* = 8.55 × 10^−03^), and colforsin (enrichment score = 0.62 and *p* = 2.33 × 10^−02^) were found to possess anti-inflammatory effects with or without additional anti-cancer effects [46,47,48]. Another candidate, suprofen (enrichment score = 0.69 and *p* = 1.99 × 10^−02^), reportedly reduces the symptoms of adjuvant arthritis, including joint inflammation and impaired growth in arthritis model rats [49]. Lycorine (enrichment score = 0.65 and *p* = 1.40 × 10^−02^) has a therapeutic effect on osteoarthritis, gout, and other rheumatic autoimmune diseases [50]. Vincamine (enrichment score = 0.53 and *p* = 4.21 × 10^−02^) was previously shown to prevent inflammation induced by lipopolysaccharide in a human corneal epithelial cell line [51]. A synthetic derivative of vincamine was suggested to have potent anti-arthritic and anti-inflammatory effects in rat models [52]. The other drug candidates that were indirectly linked to rheumatic diseases were racecadotril (enrichment score = 0.67 and *p* = 2.47 × 10^−02^) and streptozocin (enrichment score = 0.65 and *p* = 3.34 × 10^−02^). Racecadotril (enrichment score = 0.67 and *p* = 2.47 × 10^−02^) is a commonly used treatment for children with acute diarrhea indirectly linked to inflammatory diseases by overactive immune responses [53,54]. Streptozocin (enrichment score = 0.65 and *p* = 3.34 × 10^−02^) was suggested as an antibiotic and an anti-cancer drug [55,56]. Because 8 out of 10 bioactive molecules for sJIA are reported to be directly associated with inflammation or rheumatic disease, we suggest that the 10 novel drug candidates detected by CMap may, at least in part, be potential therapeutic options for sJIA.

**Figure 5:**
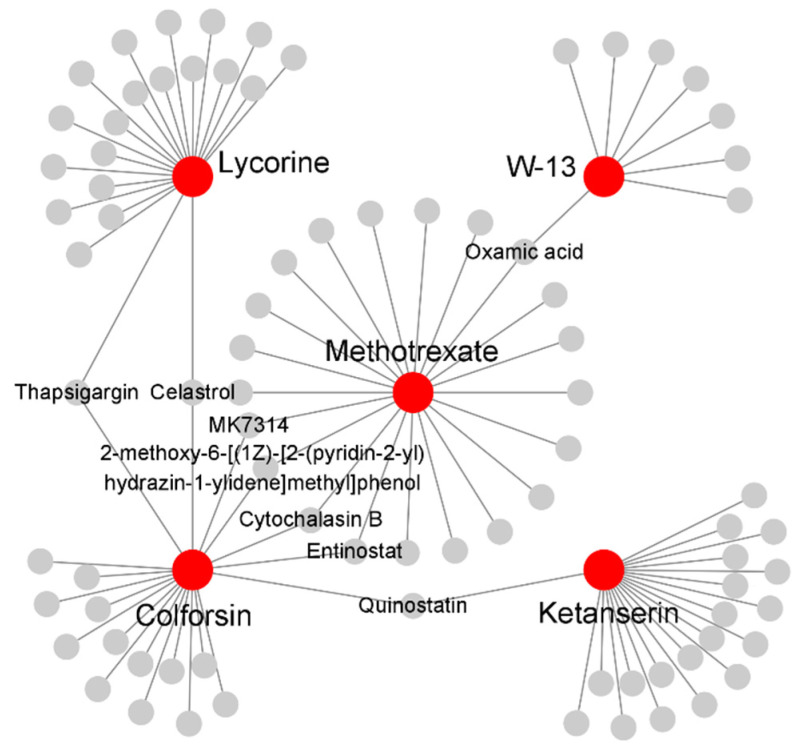
Drug interactive network between methotrexate and potential drug candidates for sJIA. Drug candidates were connected to methotrexate, the reference node, with up to 2 stopover neighboring nodes on their way. Methotrexate and the drug candidates are represented by red nodes and their neighboring drugs are displayed with grey circles. Only the drug candidates and the stopover neighbors en route to the reference node were labeled on the network.

To further determine whether the potential drug candidates are associated with the currently used drugs for sJIA, a drug interactive network was constructed using our drug candidates and methotrexate, an immunosuppressant with anti-inflammatory effects, by mode of action by network analysis (MANTRA) (Figure S7) [57,58]. Methotrexate was used as a reference node, and we found that our 4 drug candidates, W-13, ketanserin, lycorine, and colforsin, were connected to methotrexate with up to 2 stopover neighboring nodes on their routes (Figure 5). It is worth mentioning that W-13 with the highest enrichment score in CMap was directly connected to methotrexate by one stopover in MANTRA. Colforsin was also directly connected to the reference node. Ketanserin and lycorine were indirectly connected to methotrexate by 2 stopover neighbors. Overall, 8 of the 10 potentially novel drug candidates have already been implicated in inflammation and/or rheumatic diseases, and 4 out of the 8 candidates were predicted to be directly or indirectly associated with methotrexate in terms of their mechanisms of actions (MOAs) in this study, suggesting that our drug candidates may, at least in part, have potential therapeutic effects on sJIA.

## 3. Discussion

To understand the complex pathogenesis of sJIA and the expression profiles of the co-expressed gene sets in sJIA, we identified the meta-signature first using meta-analysis and then co-expression modules detected by WGCNA (Figure 1 and Figure 2). Given the large scale of the microarray data accumulated over the decades, microarray datasets are regarded as valuable resources for research [59]. Because the RNA-seq dataset provides advantages for more accurate quantification of gene expression than microarray data, it was used as a validation set for both analyses (Table 1) [59]. Then, we conducted the meta-analysis and WGCNA using microarray datasets complemented with the results of the RNA-seq dataset. The transcriptional profiling using microarrays is usually in accordance with that using RNA-seq technologies, but it also exhibits some discordance in the extent of differential gene expression [59,60]. To enhance the robustness of the results from the meta-analysis, we defined 260 DEGs that were consistently identified as up- or down-regulated genes in the microarray and RNA-seq dataset as the meta-signature. For WGCNA, we used a signed WGCNA contemplating only positive correlations between genes, since previous studies reported that this approach can detect more biologically meaningful modules than unsigned WGCNA [13,61].

Using the robust results derived from the combination of meta-analysis and WGCNA, we identified the sJIA signature that comprises 2 co-expressed gene sets respectively consisting of 103 up-regulated DEGs and 25 down-regulated DEGs (Figure 2). Up-regulated genes of the sJIA signature were enriched in innate immune processes and pathways related to producing red blood cells (Figure 3A). Among the 103 up-regulated genes, the most significant gene cluster was detected by PPI network analysis and the seed node of the cluster was *DEFA4*, which encodes a member of the defensin family of antimicrobial and cytotoxic peptides involved in host defense (Figure 4A) [33]. Based on the GO database, each gene of the cluster, including *DEFA4*, was identified as involved in neutrophil degranulation associated with innate immune processes [27,62]. *ORM1*, which encodes an acute phase plasma protein, may be one of the up-regulated novel risk genes in sJIA patients [63]. Although the function of this protein is predicted to be related to neutrophil degranulation, it has not yet been completely determined [64]. Previous studies also suggested that neutrophils play significant roles in sJIA, especially in the early inflammatory phase of the disease [9,65]. Together, the results suggest that this cluster may act as a key player in the pathogenesis and etiology of sJIA, attributed to the dysregulation of neutrophil degranulation.

Of the 128 sJIA signature genes, the 25 down-regulated genes were involved in cellular signaling pathways (Figure 3B). Among the down-regulated genes, the most significant gene cluster was identified by PPI network analysis and the seed node was *GZMB* (Figure 4B). GZMB is secreted by natural killer cells and undergoes post-translational modification to produce the active protease that induces target cell apoptosis [66]. All of the genes in the most significant down-regulated cluster, *GZMB*, *KLRG1*, *IL2RB*, and *TBX21*, were reportedly associated with natural killer cells [66,67,68,69]. TBX21, encoded by the potentially novel risk gene in this cluster, was implicated in the impaired development of natural killer cells when absent [69,70]. Avau et al. described that the defective cytotoxic capacity of natural killer cells has been detected in sJIA patients and may be involved in the underlying pathogenesis of MAS [71]. Given that, we believe that this down-regulated gene cluster in sJIA may have a shared genetic contribution to MAS.

Our study also identified drug candidates targeting the most significant up- and down-regulated clusters of the sJIA signature by using CMap (Table 2). W-13, a potentially novel drug candidate for sJIA, is regarded as an antagonist of calmodulin [43]. It was reported that calmodulin activity is involved in the various functions of neutrophils and that calmodulin inhibitors block neutrophil degranulation [72,73]. Thus, W-13 may be associated with neutrophil degranulation, suggesting that it may lead to effective therapies for sJIA. Other candidates connected to methotrexate, such as ketanserin, lycorine, and colforsin, are reported to have anti-inflammatory effects, which may also be effective for sJIA (Figure 5). Overall, our results suggest that these potential drug candidates may be used for the development of new therapeutic options for sJIA.

**Figure 6:**
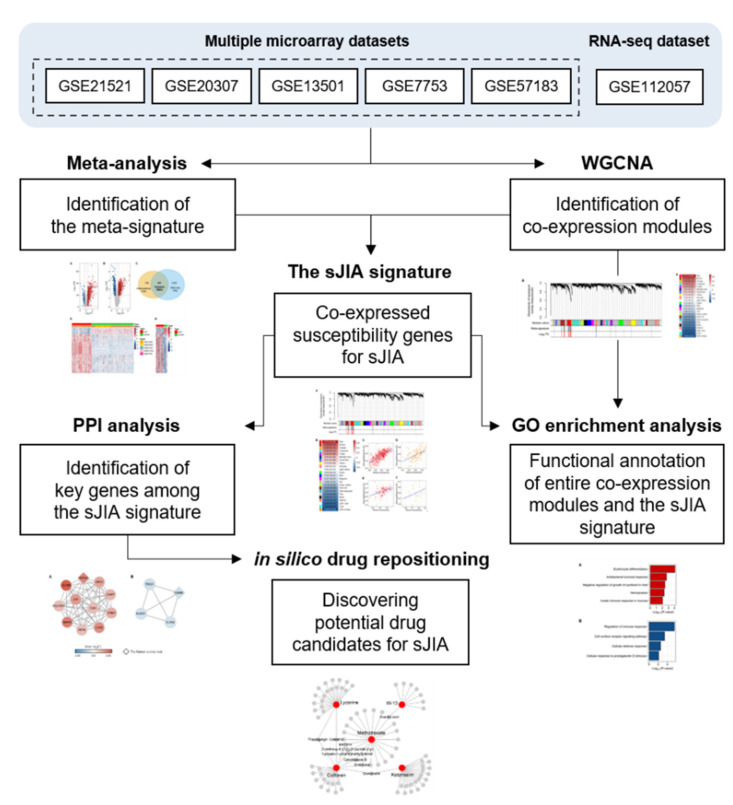
The summary of an integrative transcriptomic analysis of sJIA. sJIA, systemic juvenile idiopathic arthritis; WGCNA, weighted gene co-expression network analysis; PPI, protein-protein interaction; GO, gene ontology.

An integrative transcriptomic analysis can be a powerful approach to identify transcriptomic risk factors associated with the pathogenesis of a specific disease by increased statistical power due to a larger sample size than a single study (Figure 6) [74]. In addition, our study can detect robust susceptibility genes highly interconnected in a gene set via integrating 2 different methodologies, meta-analysis, and WGCNA. Since sJIA is a rare subtype of JIA, there have been substantial challenges in obtaining sufficient disease samples, studying its complex pathogenesis, and developing new therapeutic options. Given that, it is useful to apply our integrative analysis and in silico drug repositioning analysis together on the sJIA research to understand its pathogenesis comprehensively.

While this study identified potentially novel genetic markers and drug candidates for sJIA, we were not able to determine the causality of these genes to sJIA risk, because there were very few publicly available data to perform genome-wide association study (GWAS) or expression quantitative trait loci (eQTL) analysis. In addition, their actual genetic effects under physiological conditions could not be fully elucidated, since the results were obtained using only in silico analyses. Further functional studies may be necessary to comprehensively validate the contribution of potential biomarkers and drug candidates to the pathogenesis of sJIA. Because our study was focused only on the blood tissue, extensive research on various affected tissues is also warranted to validate our findings. Despite these limitations, we believe that our combination of meta-analysis and co-expression network analysis successfully identified a robust genetic signature of sJIA. The potential drug candidates targeting genetic markers obtained from the sJIA signature can provide novel insights into the etiology of sJIA and may contribute to the development of new therapeutic options for sJIA.

## 4. Materials and Methods

### 4.1. Data Collection

Microarray and RNA-seq datasets from PBMCs that contain mRNA expression data on human sJIA and healthy control subjects were retrieved from the NCBI GEO. Because sJIA is considered to be an autoinflammatory disease associated with immune cells, microarray and RNA-seq datasets obtained from whole blood were also collected. The selected datasets are shown in Table 1. As the datasets contained other subtypes of JIA or other auto-immune diseases, only sJIA samples were used from the collected datasets.

### 4.2. Data Preprocessing and Meta-Analysis

The preprocessing of the Affymetrix microarray datasets was conducted using the oligo R package and normalization of each dataset was performed by the robust multiarray average (rma) method [75,76,77,78]. For the Illumina dataset (GSE57183), the preprocessing and the normalization were carried out using the limma R package [79]. Subsequently, all microarray datasets were merged using the corresponding Entrez IDs of the probes and were adjusted for batch effects using the ComBat function in the sva R package [17,18]. A meta-analysis was conducted on the merged dataset using the limma R package to compute the false discovery rate (FDR) and log_2_ (fold change) (log_2_FC), which were used to identify DEGs [79]. For the RNA-seq dataset (GSE112057), the preprocessing and normalization of the raw count data was performed using the limma R package, and the identification of DEGs was carried out using the DESeq2 R package [24,79,80].

### 4.3. Weighted Gene Co-Expression Network Analysis

A signed WGCNA was applied to the merged microarray dataset of sJIA to identify co-expression modules of positively correlated genes [16]. A similarity matrix was calculated based on Pearson’s correlation between all paired genes and was converted into an adjacency matrix by the soft-thresholding method. The soft-thresholding power (β) was selected by a scale-free topology analysis. The adjacency matrix was transformed into a topological overlap matrix accounting for the similarity of genes in the co-expression network. Co-expression modules were determined by cutting a hierarchical cluster tree using a hybrid tree-cut method [16]. The summary expression profile of each module was characterized by module eigengene (ME). The minimum size of each module was 100 genes and modules with high ME correlations (r > 0.85) were merged. With the co-expression modules, a module preservation analysis was performed using the merged microarray dataset as a reference set and the RNA-seq dataset as a test set [25]. This analysis was permuted up to 200 times and Z-summary scores were calculated to identify the preserved modules.

### 4.4. Module Enrichment Analysis

To identify modules significantly associated with sJIA, the DEGs simultaneously obtained from the meta-analysis and the RNA-seq dataset were mapped onto the co-expression modules. The enrichment analysis of the common DEGs in each module was carried out using a two-sided Fisher’s exact test. The correlation between the ME of each module and the disease status was estimated using the WGCNA results with Pearson’s correlation test. The most highly-correlated modules with sJIA, enriched with up- or down-regulated common DEGs, were selected as representative modules of sJIA for subsequent steps. For each gene in the modules, Pearson’s correlation test was performed between the correlation of a gene to sJIA and that to ME of the modules. To verify the representability of the common DEGs in the modules, the abovementioned procedure was performed and the results were compared.

### 4.5. Functional Annotation of the sJIA Signature

The common DEGs included in the representative modules were regarded as genes of the sJIA signature. Up- and down-regulated genes of the sJIA signature were subjected to functional enrichment analysis, respectively. DAVID was used to conduct functional annotation of the signature [81]. Gene sets from GO BP, retrieved from the molecular signatures database (MSigDB v7.0), were used as gene sets for annotation [27,82].

### 4.6. Network Analysis and Finding Significant Clusters for sJIA

The proteins encoded by the sJIA signature were used as nodes of the PPI network. Edges between the nodes were added by using STRING [31]. The confidence level of the edges was adjusted to 0.7, and only the nodes connected to the other nodes were displayed. This network was visualized by Cytoscape (v3.7.1) and the identification of highly-interconnected regions (clusters) was performed by MCODE [32,83].

### 4.7. Identification of Drug Candidates

To identify drug candidates for sJIA, in silico drug repositioning was performed by CMap (build 02) using the up- and down-regulated gene clusters with the highest rank score in the network analysis [42]. CMap contains a number of drug signatures, which are derived from 5 cell types treated with bioactive small molecules, also known as perturbagens, at various concentrations and exposure times [42]. To unify the input signatures to probe IDs from the database of CMap, the format of the up- or down-regulated genes were converted into probe IDs in the HG-U133A platform. The enrichment score and *p*-value were computed for each perturbagen. Then, a drug network was constructed using a computational tool, MANTRA (v2.0) [57]. The resulting significant perturbagens (*p* < 0.05) and a representative medication for sJIA, methotrexate, were used as input nodes for the drug network, and the neighboring nodes were produced by MANTRA. Methotrexate was defined as the reference node of the network. The edges between the nodes were added based on the pairwise distances of MOAs between drugs by MANTRA [57]. The neighbors and the number of maximum nodes were adjusted to 20 and 500, respectively. The drugs connected to methotrexate with up to 2 stopover neighbors on their routes were visualized using Cytoscape (v3.7.1) [83].

## Figures and Tables

**Table 2:** Bioactive small molecules detected by CMap as potential drug candidates (*p* < 0.05).

CMap Name	PubChem Name	PubChem CID	Enrichment Score	*p*-Value
W-13	*N*-(4-Aminobutyl)-5-chloronaphthalene-2-sulfonamide	4299	0.95	5.77 × 10^−03^
Ketanserin	Ketanserin	3822	0.79	3.94 × 10^−03^
Gelsemine	Gelsemine	279057	0.77	5.61 × 10^−03^
Lobeline	Lobeline	101616	0.74	8.55 × 10^−03^
Suprofen	Suprofen	5359	0.69	1.99 × 10^−02^
Racecadotril	Racecadotril	107751	0.67	2.47 × 10^−02^
Streptozocin	Streptozocin	29327	0.65	3.34 × 10^−02^
Lycorine	Lycorine	72378	0.65	1.40 × 10^−02^
Colforsin	Forskolin	47936	0.62	2.33 × 10^−02^
Vincamine	Vincamine	15376	0.53	4.21 × 10^−02^

## Data Availability

Data sharing not applicable.

## Supplementary Materials

The following are available online at https://www.mdpi.com/1422-0067/22/2/712/s1, Figure S1: Scatter plots showing the results of principal component analysis (PCA), visualized by (A) disease states (control vs disease), (B) tissue sources (whole blood vs PBMC), and (C) array platform (Affymetrix vs Illumina). The x-axis and y-axis represent principal component 1 (PC1) and principal component 2 (PC2), respectively. The percentages of data variability explained by PC1 and PC2 were respectively labeled on the x- and y-axes. The color of dots represents the characteristics of each sample (green: control and red: sJIA; gray: PBMC and navy: whole blood; yellow: Affymetrix and sky-blue: Illumina Beadchip), Figure S2: A Venn diagram showing the overlap between genes discovered by at least one single study and the meta-analysis (FDR < 0.05), Figure S3: Identification of soft-thresholding power for co-expression network construction. Analyses of (A) scale-free topology and (B) the mean connectivity for selecting an appropriate soft-thresholding power, Figure S4: The result of module preservation analysis applied to the RNA-seq dataset. The green and red dashed lines indicate the thresholds of significance, Z summary score > 2 and Z summary score > 10, respectively, Figure S5: The number of genes from the meta-signature and non-meta-signature in each module. The number of genes from the meta-signature and non-meta-signature in (A) red, (B) brown, and (C) dark orange modules were labeled on the plot, Figure S6: A PPI network construction using the sJIA signature. The nodes comprise the genes of the sJIA signature. The red and blue nodes indicate up- and down-regulated genes from the red and dark orange modules, respectively. The color of the nodes corresponds to the degree of the log2FC scores. The node size corresponds to the degree of the node, Figure S7: A drug interactive network using all the 10 potential drug candidates of sJIA and methotrexate as the reference node. Triangles represent the potential drug candidates of sJIA and circles indicate their neighboring nodes. The nodes belonging to the same drug community are represented by the same color. The edges were added by the similarities between the drugs, Table S1: The list of 13 significant genes that were detected by the meta-analysis but not by single studies, Table S2: The list of 260 meta-signature genes, Table S3: The list of Entrez IDs of genes in 23 co-expression modules, Table S4: The list of genes in sJIA signature. Mean log2FC indicates the mean value of log2FC from the merged microarray dataset and RNA-seq dataset, Table S5: Functional annotation of 23 co-expression modules using GO terms. GO terms significantly associated with each module were displayed in each sheet of this file (p < 0.01), Table S6: The up-regulated GO terms in sJIA, Table S7: The down-regulated GO terms in sJIA, Table S8: The information on 66 nodes in the PPI network.

## Abbreviations

JIA	Juvenile Idiopathic Arthritis
sJIA	Systemic Juvenile Idiopathic Arthritis
WGCNA	Weighted Gene Co-expression Network Analysis
MAS	Macrophage Activation Syndrome
DEG	Differentially Expressed Gene
*IL-1*	*Interleukin 1*
*IL-6*	*Interleukin 6*
*TNF-α*	*Tumor necrosis factor-α*
NCBI	National Center for Biotechnology Information
GEO	Gene Expression Omnibus
PCA	Principal Component Analysis
FDR	False Discovery Rate
Log_2_FC	Log_2_(Fold Change)
NGS	Next Generation Sequencing
RNA-seq	RNA-sequencing
PBMC	Peripheral Blood Mononuclear Cell
ME	Module Eigengene
GO BP	Gene Ontology Biological Process
DAVID	Database for Annotation, Visualization, and Integrated Discovery
PPI	Protein–Protein Interaction
STRING	Search Tool for the Retrieval of Interacting Genes
MCODE	Molecular Complex Detection
*DEFA4*	*Defensin alpha 4*
*OLFM4*	*Olfactomedin 4*
*ORM1*	*Orosomucoid 1*
*TCN1*	*Transcobalamin 1*
*GZMB*	*Granzyme B*
KLR	Killer cell lectin-like receptor
*KLRG1*	*Killer cell lectin-like receptor G1*
*IL2RB*	*Interleukin 2 receptor subunit beta*
*TBX21*	*T-box transcription factor 21*
CMap	Connectivity Map
W-13	N-(4-aminobutyl)-5-chloronaphthalene-2-sulfonamide
MANTRA	Mode of Action by Network Analysis
MOAs	Mechanisms of Actions
GWAS	Genome-Wide Association Study
eQTL	Expression Quantitative Trait Loci
MSigDB	Molecular Signatures DataBase
