# Supplementary material for: An Integrative Transcriptomic Analysis of Systemic Juvenile Idiopathic Arthritis for Identifying Potential Genetic Markers and Drug Candidates
Source: Int J Mol Sci. 2021 Jan 12;22(2):712. doi: 10.3390/ijms22020712 (PMC7828236; doi:10.3390/ijms22020712)
Supplement: Supplementary file 1 [file ijms-22-00712-s001.zip › sJIA_Supplementary_Figure/sJIA_suppl_figure.pdf]

Supplementary Figure S1

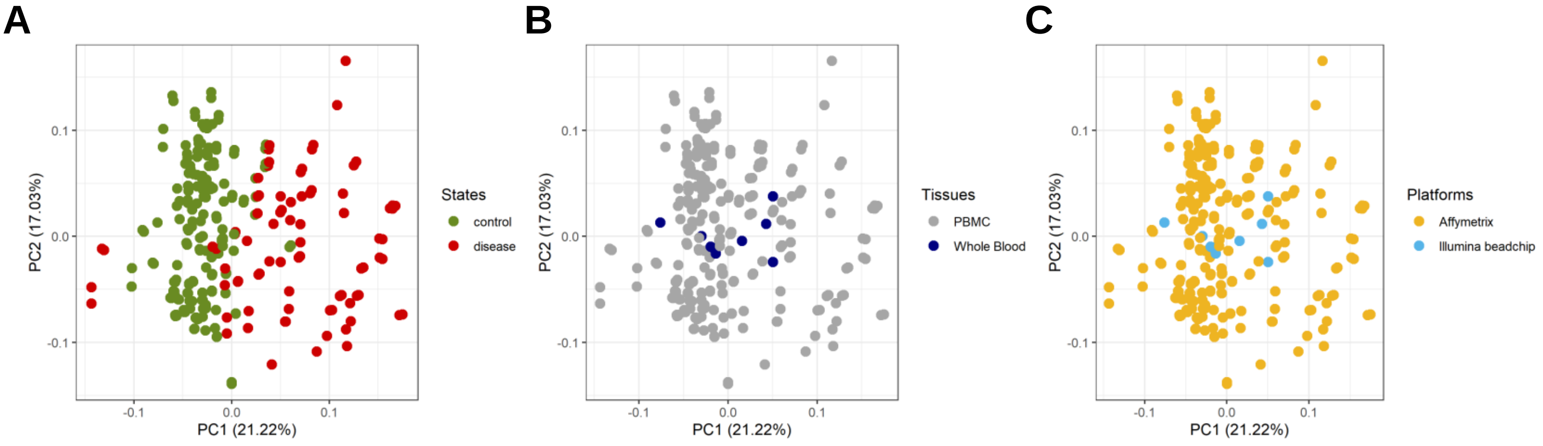

Supplementary Figure S1. Scatter plots showing the results of principal component analysis (PCA), visualized by (**A**) disease states (control vs disease), (**B**) tissue sources (whole blood vs PBMC), and (**C**) array platform (Affymetrix vs Illumina). The x-axis and y-axis represent principal component 1 (PC1) and principal component 2 (PC2), respectively. The percentages of data variability explained by PC1 and PC2 were respectively labeled on the x- and y-axes. The color of dots represents the characteristics of each sample (green: control and red: sJIA; gray: PBMC and navy: whole blood; yellow: Affymetrix and sky-blue: Illumina Beadchip).

Supplementary Figure S2

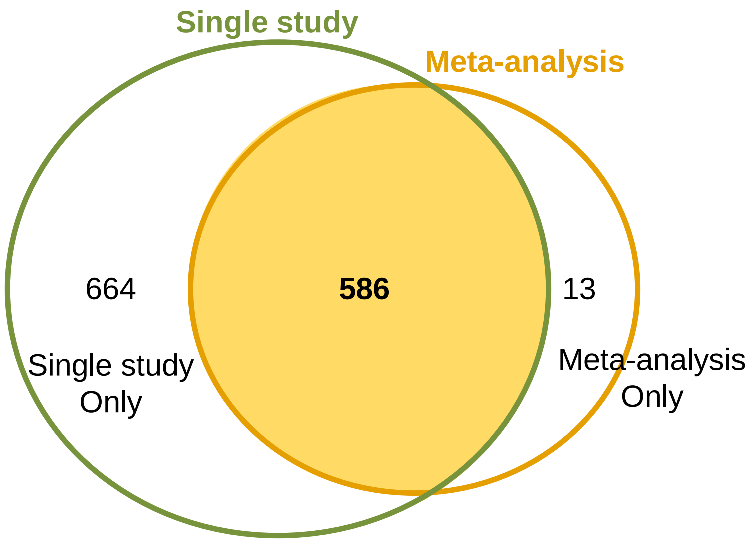

Supplementary Figure S2. A Venn diagram showing the overlap between genes discovered by at least one single study and the meta-analysis (FDR < 0.05).

Supplementary Figure S3

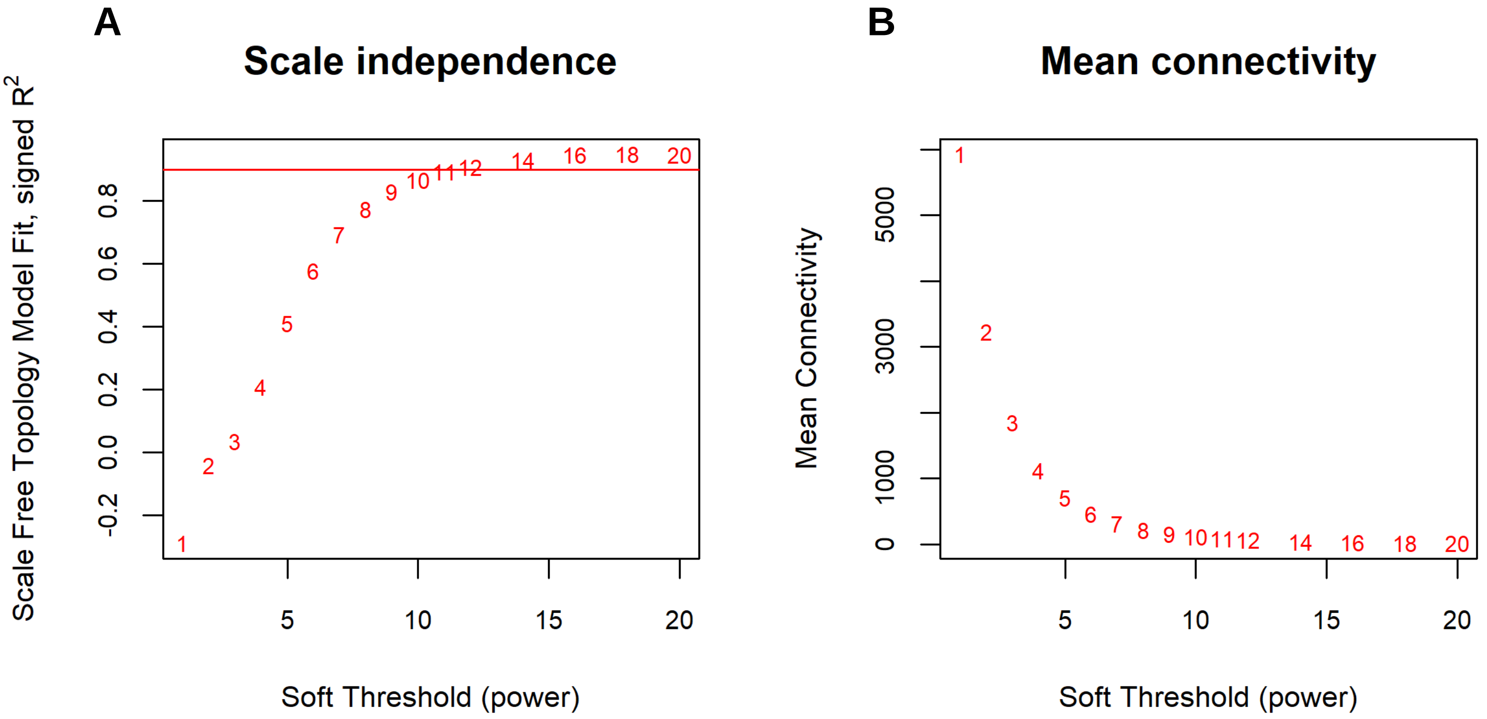

Supplementary Figure S3. Identification of soft-thresholding power for co-expression network construction. Analyses of (**A**) scale-free topology and (**B**) the mean connectivity for selecting an appropriate soft-thresholding power.

Supplementary Figure S4

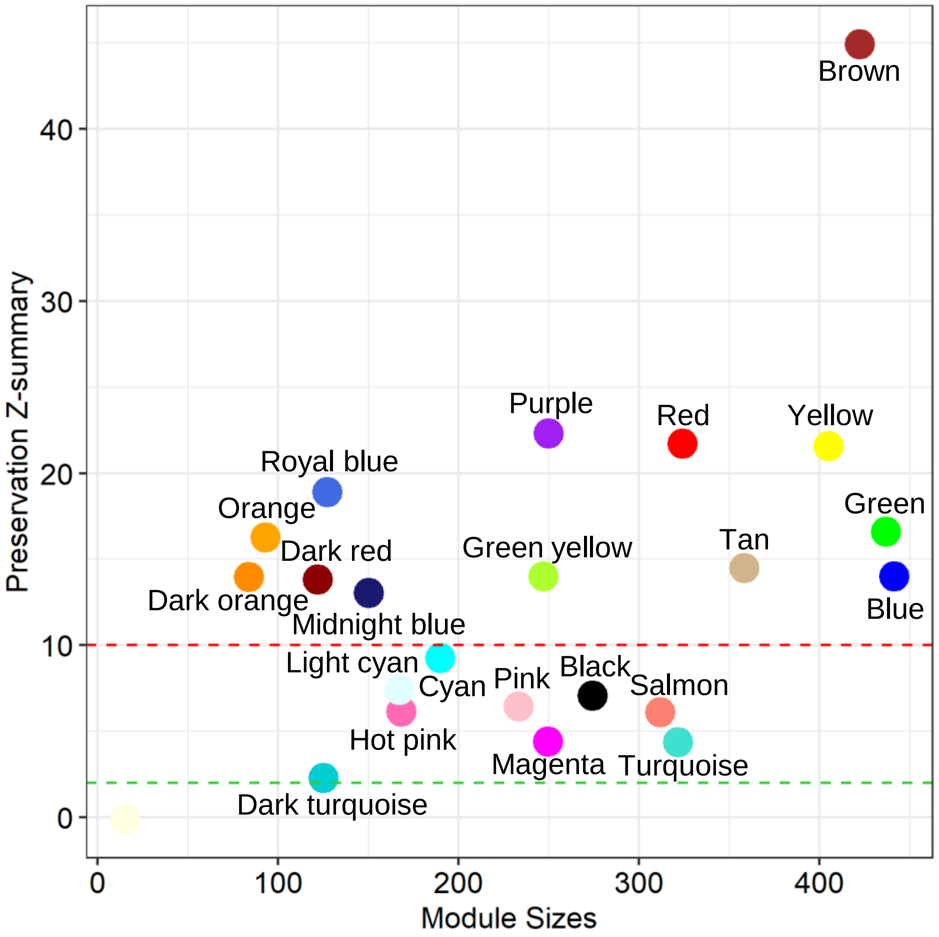

Supplementary Figure S4. The result of module preservation analysis applied to the RNA-seq dataset. The green and red dashed lines indicate the thresholds of significance, Z summary score > 2 and Z summary score > 10, respectively.

Supplementary Figure S5

A

	Meta-signature	Non Meta-signature
Red module	103	303
Non red module	158	11027

B

	Meta-signature	Non Meta-
Brown module	73	381
Non brown module	188	11142

C

	Meta-signature	Non Meta-signature
Dark orange module	26	85
Non dark orange module	235	11245

Supplementary Figure S5. The number of genes from the meta-signature and non-meta-signature in each module. The number of genes from the meta-signature and non-meta-signature in (**A**) red, (**B**) brown, and (**C**) dark orange modules were labeled on the plot.

Supplementary Figure S6

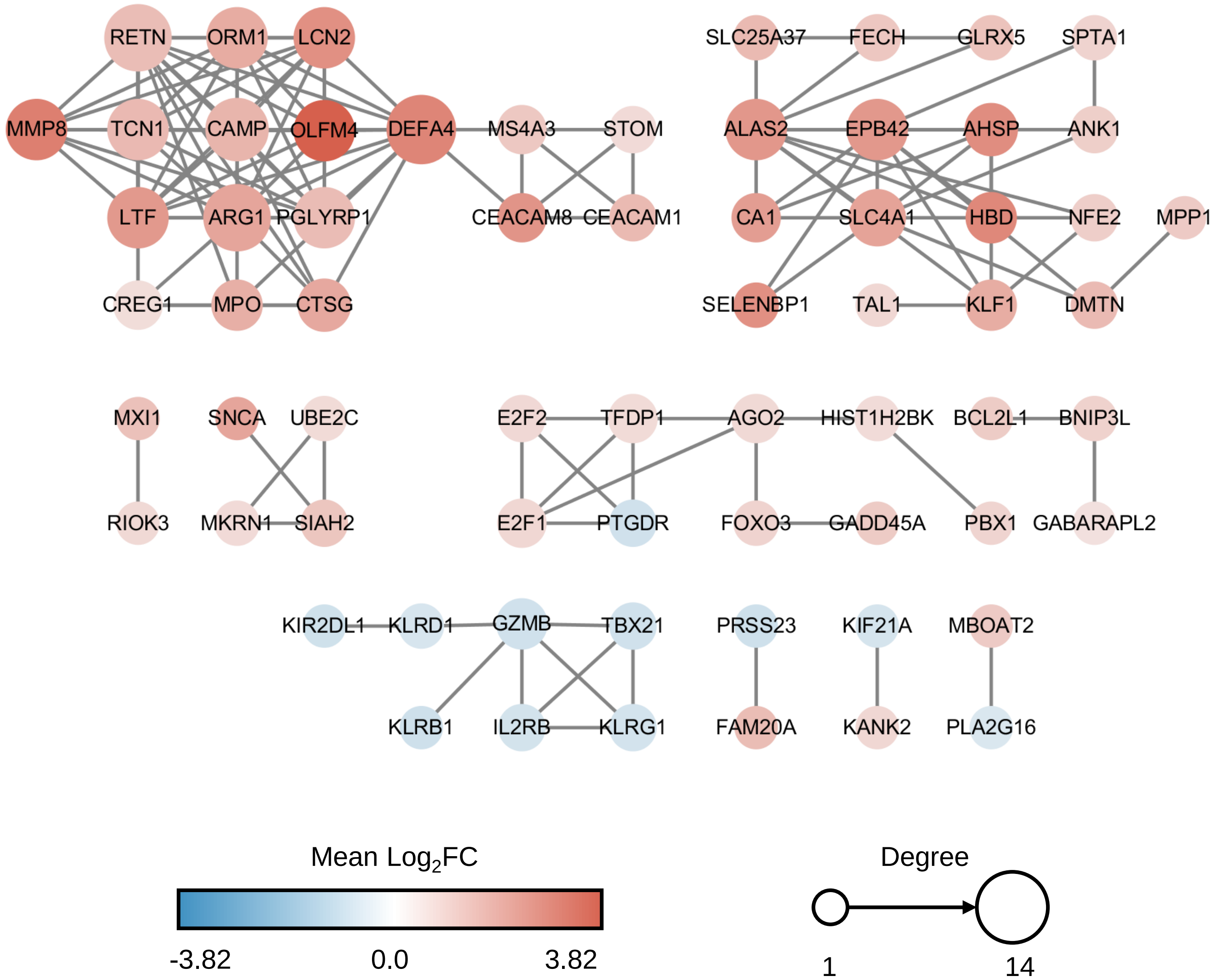

Supplementary Figure S6. A PPI network construction using the sJIA signature. The nodes comprise the genes of the sJIA signature. The red and blue nodes indicate up- and down-regulated genes from the red and dark orange modules, respectively. The color of the nodes corresponds to the degree of the log_2FC scores. The node size corresponds to the degree of the node.

Supplementary Figure S7

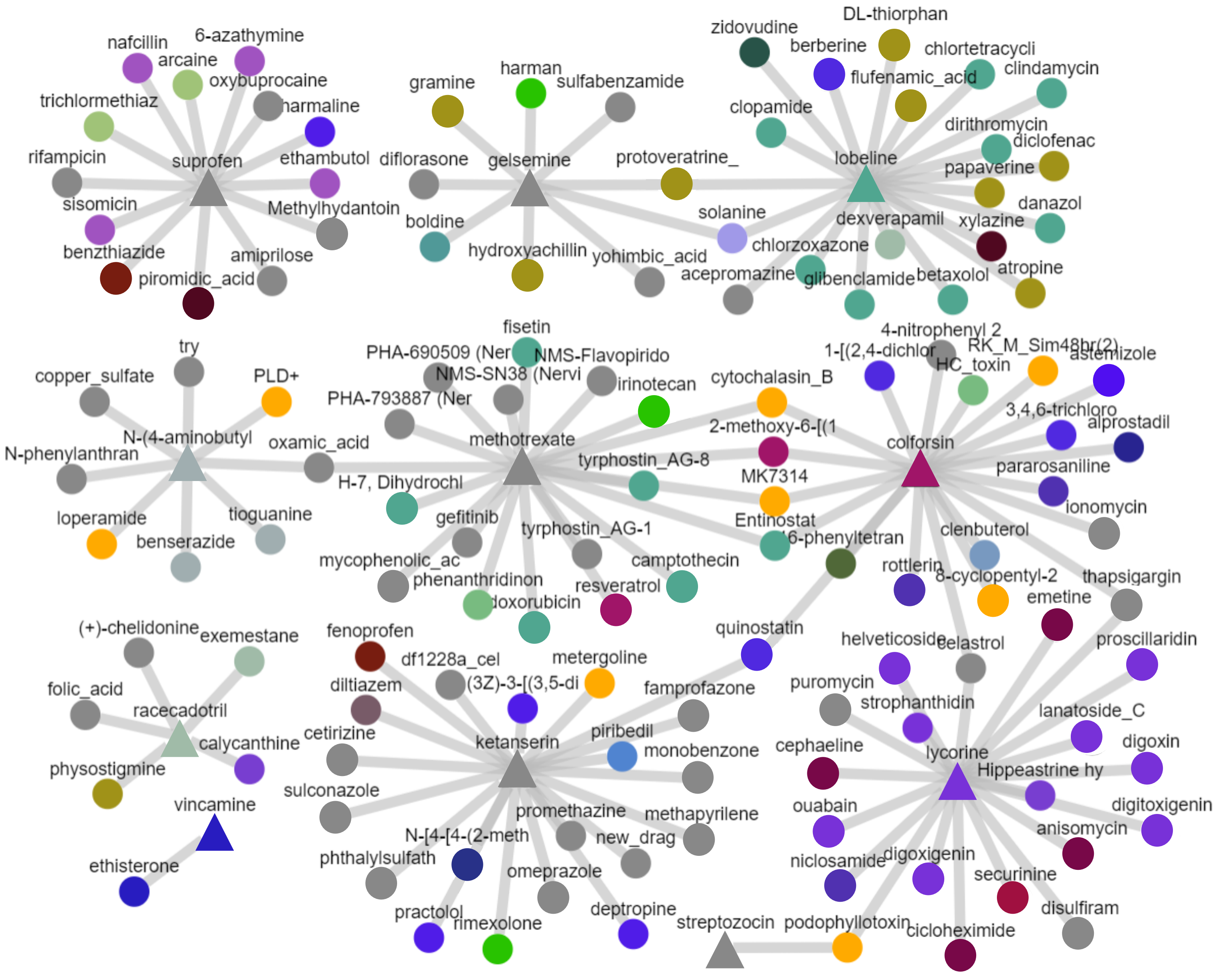

Supplementary Figure S7. A drug interactive network using all the 10 potential drug candidates of sJIA and methotrexate as the reference node. Triangles represent the potential drug candidates of sJIA and circles indicate their neighboring nodes. The nodes belonging to the same drug community are represented by the same color. The edges were added by the similarities between the drugs.
